# Interspecific Resource Competition Effects on Fisheries Revenue

**DOI:** 10.1371/journal.pone.0053352

**Published:** 2012-12-28

**Authors:** Karen E. van de Wolfshaar, Tim Schellekens, Jan-Jaap Poos, Tobias van Kooten

**Affiliations:** 1 Department Fish, Institute for Marine Resource and Ecosystem Studies (IMARES), Wageningen UR IJmuiden, The Netherlands; 2 Department Delta, Institute for Marine Resource and Ecosystem Studies (IMARES), Wageningen UR, Yerseke, The Netherlands; University of Kent, United Kingdom

## Abstract

In many fisheries multiple species are simultaneously caught while stock assessments and fishing quota are defined at species level. Yet species caught together often share habitat and resources, resulting in interspecific resource competition. The consequences of resource competition on population dynamics and revenue of simultaneously harvested species has received little attention due to the historical single stock approach in fisheries management. Here we present the results of a modelling study on the interaction between resource competition of sole (*Solea solea*) and slaice (*Pleuronectus platessa*) and simultaneous harvesting of these species, using a stage-structured population model. Three resources were included of which one is shared with a varied competition intensity. We find that plaice is the better competitor of the two species and adult plaice are more abundant than adult sole. When competition is high sole population biomass increases with increasing fishing effort prior to plaice extinction. As a result of this increase in the sole population, the revenue of the stocks combined as function of effort becomes bimodal with increasing resource competition. When considering a single stock quota for sole, its recovery with increasing effort may result in even more fishing effort that would drive the plaice population to extinction. When sole and plaice compete for resources the highest revenue is obtained at effort levels at which plaice is extinct. Ignoring resource competition promotes overfishing due to increasing stock of one species prior to extinction of the other species. Consequently, efforts to mitigate the decline in one species will not be effective if increased stock in the other species leads to increased quota. If a species is to be protected against extinction, management should not only be directed at this one species, but all species that compete with it for resource as well.

## Introduction

Many fisheries harvest multiple species simultaneously as a result of indiscriminate fishing methods and spatiotemporal mixing of fish species. An unavoidable aspect of these fisheries is that fishing mortalities imposed on the targeted species are interrelated: it is impossible to fish one species, while sparing another. However, differences in ecology and life history between species cause these simultaneously caught species to respond differently to fishing pressure. Optimally exploiting one species may lead to overexploitation of another, while sparing a sensitive species may mean leaving valuable resources unused. The effects of these two aspects of marine resource exploitation; the interspecific correlations in fishing mortality resulting from indiscriminate fisheries (e.g. [1]), and the ecological interactions between species (e.g. [2]), have both been studied extensively in isolation. Where marine resource exploitation has been studied with these combined effects, the focus has been on predator-prey relationships (e.g. [3]) thereby neglecting interspecific resource competition (but see [4]). Here we combine the two by studying how simultaneously caught populations respond to fishing whilst competing for limited food.

When two species compete for limited food, their population dynamics are interdependent. Simply put, if one species is abundant, and consumes the available food, growth and reproduction of individuals of the other species is reduced. Ultimately, the abundance of one species will decline, resulting in competitive exclusion. Mortality on the strongest competitor can thereby help species that might otherwise lose through interspecific competition [5]. Increased fishing mortality on one species may thus actually positively affect the growth, reproduction and ultimately the abundance of its competitor [6]. In this setting, the fishery acts as a top predator on the competing species. This has important implications for management of natural resources: reducing mortality of one species leading to increases in abundance, can hamper the recovery of other species and limit their abundance.

One example of a multispecies fishery is the beam trawl fishery in the North Sea [7], [8]. In this fishery, a trawl is pulled over the seabed, and a fraction of the fish in the trawl path is caught in the net. This fishing method is highly indiscriminate, catching many species of fish but also benthic macro-invertebrates [9]. The main target species for the beam trawl fleet in the North Sea are plaice (*Pleuronectes platessa*) and sole (*Solea solea*). The price of sole is almost 10-fold the price of plaice. Management regulations have led to substantial discarding of small individuals of especially plaice. As a consequence, fishing mortalities have often been higher than indicated by landings alone [10], [11].

There is evidence that the two main target species of the Dutch beam trawl fleet, sole and plaice, compete for invertebrate resources such as polychaetes and bivalves [12]. This results in a configuration of the competition – fishery system described above, where fishing intensity has both a direct negative (fisheries mortality) and a potential indirect positive effect (decrease of competition) on each of the species. The crucial questions that we study here is: what is the net result of these two opposing mechanisms on the populations of the two species, and how does this affect the revenue of the beam trawl fishery exploiting sole and plaice stocks in the North Sea? This question is studied using a biomass-based stage-structured population model.

Our results indicate that resource competition alters expected revenue compared to a system without interspecific competition. Whether considered separately or combined, without considering interspecific competition the revenue of the species is a parabolic curve with increasing effort. When resource competition is included, the revenue of the sole population increases with increasing effort, due to increased resource availability resulting from plaice mortality. As a result of the increasing sole stock, the revenue of the stocks combined is bimodal with a local minimum. When considering a single stock quota for sole, its population recovery with increasing effort, due to a decrease in the plaice population and the subsequent decrease in resource competition, may result in even more fishing effort as the sole stock appears healthy. However, an increase in effort based on the sole population would drive the plaice population to extinction. Moreover, when resource competition is included the total highest revenue is obtained at effort levels for which plaice is extinct, partly due to the price difference between plaice and sole. Our results indicate that separate quota for species with resource overlap that co-occur in catches may lead to overfishing of the species with the highest catchability in both adult and large juvenile stages. This supports the need to include interspecific competition, in addition to the more often studied predator-prey interactions [3], in advocating combined species management.

### Model and Parameter Estimates

#### Consumer-resource model

To study the interaction between sole and plaice in terms of resource competition and fishing mortality the biomass-based size-structured population model framework introduced by De Roos *et al.* [13] was used. This model is a stage-structured approximation of a more complex physiologically structured model. Under equilibrium conditions, there is complete correspondence between the models, while the non-equilibrium dynamics of the simple model approximate that of the complex formulation [13]. The key biological features are described below, for a detailed mathematical derivation we refer to De Roos *et al.* [13].

**Figure 1:**
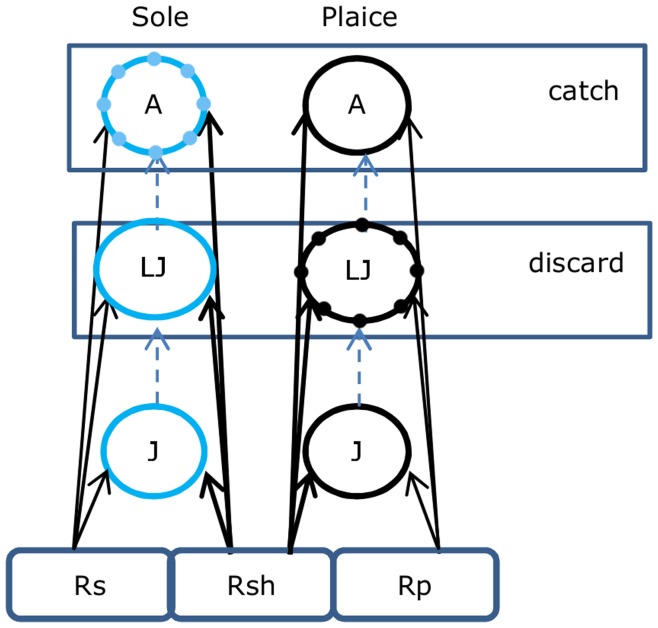
Modelled food web of sole and plaice foraging on potentially two resources. Both populations are structured into three stages: small juveniles (J), large juveniles (LJ) and adults (A). Dashed grey lines denote transitions between stages based on growth. Solid lines denote consumer-resource interaction. Both large juveniles and adults suffer from fishing mortality, but only adults makeup the catch while large juveniles are discarded. The stages with dots indicate which of the two species has the highest catchability for that particular stage.

We model two fish populations, each divided into three stages: small juveniles (*J*), large juveniles (*LJ*) and adults (*A*) (Figure 1). We use parameters for the fish species plaice (*Pleuronectes platessa*) and sole (*Solea solea*), the main targets for the North Sea beam trawl fishery. Since the equations for both species are identical we present only one set of equations, which is valid for both species.

Each consumer species *n* has an exclusive resource (*R_n_*), shared by all its life stages. In addition, there is a shared resource (*R_sh_*) which both species can consume to a variable degree (Figure 1). We assume that the resource populations follow semi-chemostat dynamics, with growth rate *r*, reaching maximum equilibrium biomass *K* when consumers (sole and plaice) are absent. The maximum equilibrium density of the exclusive plaice resource (*K_p_*) is constant, while the maximum equilibrium densities of the exclusive sole resource and the shared resource are scaled with fraction *ω* (0≤*ω*≤1) so that
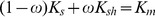
(1)


This means that when *ω* scales competition. When *ω* equals 0 the maximum equilibrium density of the sole resource equals *K_m_* and that of the shared resource equals 0 and there is no resource competition. When *ω* equals 1 sole has only the shared resource and fully competes with plaice. The maximum equilibrium density of the exclusive resources for plaice (*K_p_*) is three times *K_m_* to ensure that plaice is more abundant than sole, as is the case in the North Sea [14]. Sole and plaice stages forage following a Holling type II functional response with a half-saturation constant *R_h_* and a mass specific maximum ingestion rate *I_max_*. The total ingested resources (*IR_i,n_*) by a certain stage *i* of species *n*, depends on the the biomass densities of its exclusive (R_n_) and shared resource (R_sh_) according to:
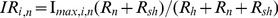
(2)


The differential equations describing the dynamics of the exclusive resource for species *n* and the shared resource *R_sh_*, eaten by sole and plaice are as follows:
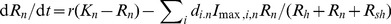
(3)

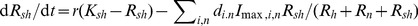
(4)With 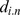 representing the density of each stage *i* of species *n* (representing J, LJ or A for both species). The ingested resource is converted with an efficiency *δ*, and this, less the species mass specific metabolic rate (*T*), then gives the net biomass production (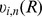) of stage *i* of species *n*:



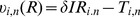
(5)The following set of differential equations then describes the dynamics of the three stages of plaice and sole:
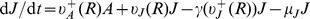
(6)

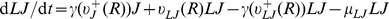
(7)

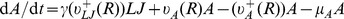
(8)Where *µ_i_* is the total mortality rate in stage *i*, 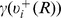 is the rate at which small juveniles and large juveniles mature into the large juvenile and adult stage, and 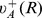 is the reproduction rate.

The maturation rate depends on the net biomass production of the stage, the size range over which individuals grow, and mortality:
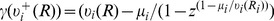
(9)


The parameter *z* is the ratio between the mass at which an individual enters the stage and at which it develops to the next stage [13]. For adults, it is assumed that all surplus energy is converted into offspring and no growth occurs. Hence, the net biomass production of the adult stage equals the biomass reproduction rate. Biomass can only be transferred between stages when the net intake is positive (i.e. 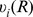
>0), to ensure that negative reproduction and maturation do not occur). If the net biomass production is negative the stage suffers from starvation mortality, and recruitment and maturation do not take place (i.e. 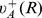 = 0, 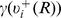 = 0). All stages suffer from background mortality *µ*, Large juveniles and adults additionally are subjected to fishing mortality.

#### Fisheries

To model the combined fisheries on sole and plaice we use a linear functional response. Sole and plaice are caught together, and hence we use a single harvesting intensity (*E*, for effort). For each species and stage, the effort is modulated by the relative catchability *f_n,i_*, the tendency of each susceptible stage to be caught in the fishing gear. Hence, for each species/stage, the fishing mortality is
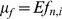
(10)


Assuming a species-independent effort, we can then use the estimated annual fishing mortality from ICES reports [14] on sole and plaice to calculate the relative catchability (Table 1). This procedure yields the best available measure for catchability, but at the same time means that effort (*E*) in our model is an arbitrary unit of harvesting intensity which cannot be quantitatively compared with measured values of effort. Because effort is an arbitrary unit, a measured cost of fishing cannot be used to calculate the net profit. We use revenue to characterise the catches, rather than simply biomass, because of the strong price asymmetry between the species. Using the revenue allows us to add up the two stocks in a way which is meaningful in relation to their exploitation. The large juvenile (*LJ*) and adult (*A*) stages of both fish species are subjected to fishing, but only adult stages are landed, while large juveniles suffer from mortality and are not included in the calculation of revenue.

**Table 1:** Model variables, parameters and their values.

Variable	Value		Unit	Description
*J*			gL^−1^	Juvenile biomass
*LJ*			gL^−1^	Large Juvenile biomass
*A*			gL^−1^	Adult biomass
*R*			gL^−1^	Resource biomass
	*Plaice*	*Sole*		
*L_J_*	1.5	0.85	cm	Size at settlement
*L_LJ_*	10	12	cm	Size at catch
*L_A_*	27	24	cm	Size at landing
*W/L J*	1.7/5.6	2.3/6.1	g cm^−1^	Average weight/lengthjuvenile
*W/L LJ*	65.5/18.5	66.4/18.0	g cm^−1^	Average weight/lengthlarge juvenile
*W/L A*	208.6/27	160.7/24	g cm^−1^	Average weight/lengthadadult
*I_maxJ_*	0.069	0.050	gg^−1^d^−1^	Maximum intakerate juvenile
*I_maxLJ_*	0.020	0.016	gg^−1^d^−1^	Maximum intakerate largejuvenile
*I_maxA_*	0.013	0.011	gg^−1^d^−1^	Maximum intakerate adult
*T*	0.0037	0.0032	gg^−1^d^−1^	Maintenancerate
*z_J-LJ_*	0.003	0.0003		Size ratio juvenileto large juvenile
*Z_LJ-A_*	0.048	0.118		Size ratio largejuvenile to adult
*Δ*	0.36	0.36		Food conversion factor
*µ*	0.001	0.001	d^−1^	Background mortality
*P*	1.38	10.06	€ kg^−1^	Average price
*f_LJ_*	5E-5	2E-6		Catchability large juveniles
*f_A_*	0.001272	0.001455		Catchability adults
*E*	Varied		d^−1^	Fishing effort
*Rh*	0.01		gL^−1^	Feeding half saturationconstant
*R*	0.1		d^−1^	Resource regrowth rate
*K_m_*	0.3		gL^−1^	Resource maximum Sole
*K_p_*	1		gL^−1^	Resource maximum Plaice
Ω	Varied			Resource maximum scalar

Using the price of adult fish biomass (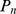) and their biomass density and multiplying with the catchability of adults 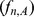 and the effort (*E*) the revenue (*Rev*, unit €/d) is calculated for each species *n*:
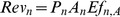
(11)


We assume that all sizes above the landing size are caught and landed.

### Parameters

Both the sole and plaice population are divided into three stages: small juveniles, large juveniles and adults. The minimum size of a juvenile is set to the size at settlement, as little is known about the pelagic fate of the larvae. For the settlement size of sole we use 0.85 cm [15], and for plaice 1.5 cm [16]. We set the boundary between the juvenile and large juvenile stage at 10 and 12 cm for plaice and sole respectively, which are the minimum sizes caught in an 80 mm mesh size beam trawl [17]. The difference between species is due to the shapes of individual fish. Sole have a relatively slender, elongated body and are hence able to escape through the net meshes at greater lengths than plaice, which are more diamond-shaped. These sizes are well below the allowed landing size and any caught biomass of large juveniles is hence discarded (put back overboard often dead). The landing size (European Council Regulation No. 850/98) is taken as the length at which individuals mature (referred to as adult-stage). The adult sizes for sole and plaice are 24 cm and 27 cm, respectively (Table 1).

To calculate the revenue, we use the average price of sole and plaice at Dutch auctions in 2009, 10.06 €kg^−1^ for sole and 1.38 €kg^−1^ for plaice [18].

To calculate the maximum ingestion rate we used the maximum surface area-specific ingestion rate for plaice (548 Jd^−1^ cm^−2^ [19]), and for sole (460 Jd^−1^ cm^−2^ [20]). The energy content of the resource polychaete worms (23 kJg^−1^) [21], was converted to wet weight using 16% as ash free dry weight to wet weight ratio [22]. The maximum ingestion rates presented in energy content were then converted to gram prey per day per cm^2^. Using the average length and weight per stage, the species- and stage-specific maximum biomass specific ingestion rate was calculated (Table 1). The average weight of a stage was calculated using a length-weight relationship of the form *W = aL^b^* (with *W* in g and *L* in cm), with for sole *a* = 0.0091, *b* = 3.077 and for plaice *a* = 0.0089, *b* = 3.0353 [23]. The start and end weight of each stage was then used to calculate the average weight (*W_avg_*) in the juvenile and sub-adult stages following the equation provided by Van Leeuwen *et al.* [24], where *W_avg_ = W_max_– W_min_/(ln W_max_ – ln W_min_)*. For the adult stage the weight at maturation was used.

Ingested food is converted into body mass using a conversion factor of 0.36, for both species. This conversion factor is calculated using energy conversions provided in the Dynamic Energy Budget theory [25], the cost for growth (7 kJg^−1^) [19], the energy content (5.6 kJg^−1^) [26] and the assimilation efficiency (0.8) [19], to obtain an overall conversion factor for all stages.

The species specific maintenance cost, given in Jd^−1^ cm^−3^ by Eichinger *et al.* [20], was converted to gg^−1^d^−1^, using the energy content of fish, 5.6 kJg^−1^ wet weight [26].

Following Van Leeuwen *et al.* [24] we assume a background mortality of 0.001 d^−1^ for all stages of all species, which approximates 70% annual survival. In addition, large juveniles and adults are subjected to fishing mortality. We used a Von Bertallanffy growth function to calculate the mean age of adults in each species. We then used age-specific annual fishing mortalities for the period 1975–2010 [14] to estimate catchability. For plaice and sole adults we used the average fishing mortality of ages ≥4 years, resulting in a daily catchability of 0.001272 d^−1^ for plaice and 0.001455 d^−1^ for sole. Estimates of plaice discard percentages varied between 18–30% (discarded biomass as a fraction of landed biomass) in 1970–1990 up to 50% in 2003. Those of sole were estimated at 3–13% in 1970–1990 and 14% in 2003 [17]. We therefore tuned the catchability of large juvenile sole and plaice (in absence of resource competition) such that a discard fraction of 0.3 for plaice and 0.1 for sole was realized in the model.

Sole and plaice are benthic predators and compete for resources. The biomass of benthic macro-invertebrates in the North Sea was estimated at 10.1 gm^−2^ in absence of fish [27]. When assuming that the benthic habitat is made up of the 10 cm of water above the sea floor and that benthic macro-invertebrates consumed are concentrated in 1/3 of the bottom surface the maximum resource density can be set to 0.3 gL^−1^. As plaice occupies a larger region in the North Sea than sole we assume the maximum resource density for plaice exclusive resource to be 1 gL^−1^, representing a larger plaice habitat. In a semi-chemostat the productivity is given by *rK,* the product of the intrinsic growth rate and the maximum resource density. Hiddink *et al.* [27] estimate the benthic productivity in the North Sea at 10.6 gm^−2^y^−1^, which yields an intrinsic growth rate of ∼0.1 d^−1^.

#### Analysis

We study the effect of resource overlap (*ω*) and harvesting effort (*E*) on discard rate and revenue of the above model following equilibrium dynamics. All equilibrium continuations are performed using MATCONT [28].

## Results

### Competition

**Figure 2:**
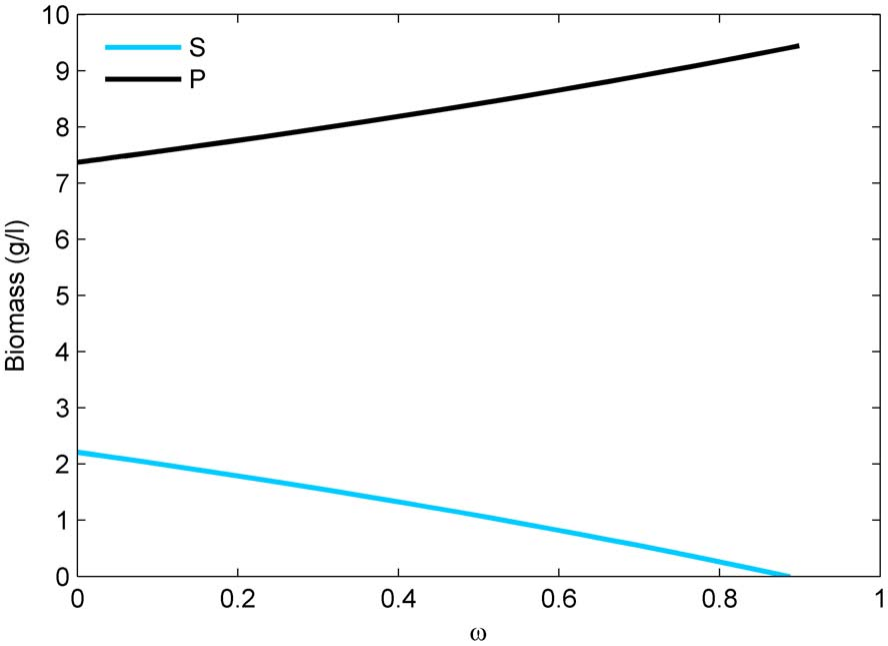
Biomass of plaice and sole as function of the resource carrying capacity scalar. With ω = 0 there is no resource competition and ω = 1 there is complete resource overlap. No fishing included.

When ω increases, sole increasingly has to share its resource with plaice, while resource availability for plaice increases. Increasing competition (*ω*) therefore results in an increase in plaice biomass (Figure 2). At near complete resource overlap the sole population is outcompeted by plaice and goes extinct (Figure 2). Coexistence of sole and plaice with resource competition is possible when sole and plaice are not limited by the shared resource and the availability of exclusive resources can sustain either species. Since the availability of exclusive resource for sole declines with resource overlap, and it is the weaker competitor for resources, an increase in resource overlap limits coexistence possibilities.

### Fishing Effort

**Figure 3:**
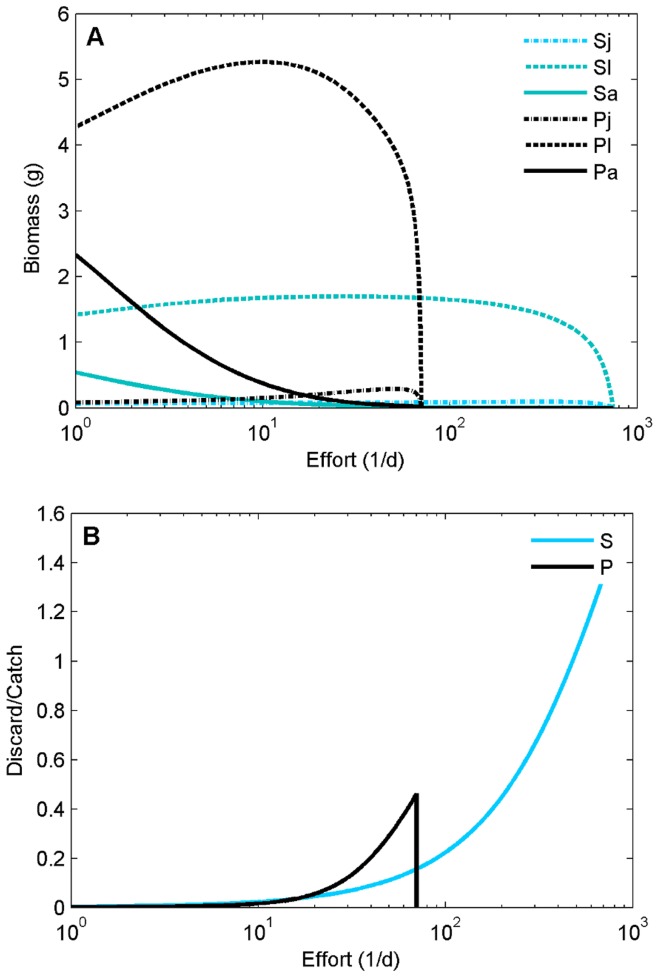
Biomass and discard over catch as function of effort. Biomasses of the species and stages as function of effort (A), and discard over catch (B). Without competition for a shared resource (ω = 0).

Without competition (*ω* = 0) the adult biomass of both species decreases exponentially with increasing harvesting effort (Figure 3 A). In contrast, the biomass of small and large juveniles of both species follows a hump-shaped trajectory with increasing effort. This pattern is the result of intraspecific competition. The higher mortality of adults decreases resource competition among adults, leading to an increased production of juveniles and an increased inflow into the large juvenile stage, which more than compensates for the additional mortality from harvesting (we refer the reader to [29] and [30] for a mechanistic explanation of these patterns). This effect is more pronounced for plaice than for sole (Figure 3). The hump-shaped response of the large juvenile equilibrium biomass to harvesting effort causes an increase in the fraction of the total catch that is discarded (Figure 3 B). The plaice population goes extinct at lower effort than the sole population (Figure 3 A). This is because in comparison with sole, a larger fraction of plaice total biomass is in the large juvenile and adult stages, so that a larger fraction of the population is susceptible to harvesting mortality. The distribution of biomass over the stages of each species is a result of species-dependent parameter values and the relative sensitivity to harvesting. It is hence an effect of physiological differences between the species. This indirect effect occurs in spite of the higher catchability of sole adults (Table 1).

**Figure 4:**
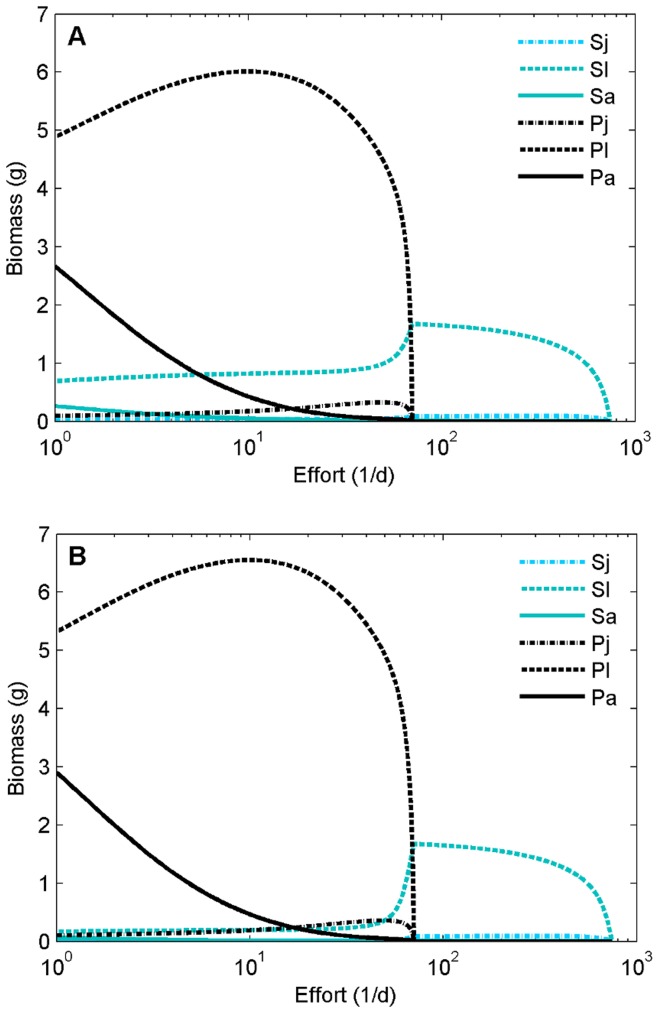
Biomasses of sole and plaice as function of effort, for different competition levels of resource competition. ω = 0.5 (A, C) and ω = 0.8 (B, D).

Higher interspecific competition for resources (*ω* >0) causes plaice to gain access to an extra resource, whereas sole must share its only resource. As a result, increasing competition increases biomass of plaice and reduces that of sole (Figure 2). Even though fishing mortality has a direct negative effect on both the target species, it affects plaice more strongly than sole because more of plaice biomass is susceptible to harvesting. As a consequence of substantial resource competition, an indirect positive effect of harvest mortality on sole occurs due to reduced interspecific competition. When harvesting mortalities increase, the effect of decrease in competition is higher than the effect of increasing mortality in sole. The net effect is that sole biomass increases, while plaice biomass decreases until plaice goes extinct. Because a decrease of competition cannot occur after extinction of plaice, only the direct negative effect of harvesting is left for sole, causing it to decrease in biomass with increasing harvesting mortality (Figure 4).

**Figure 5:**
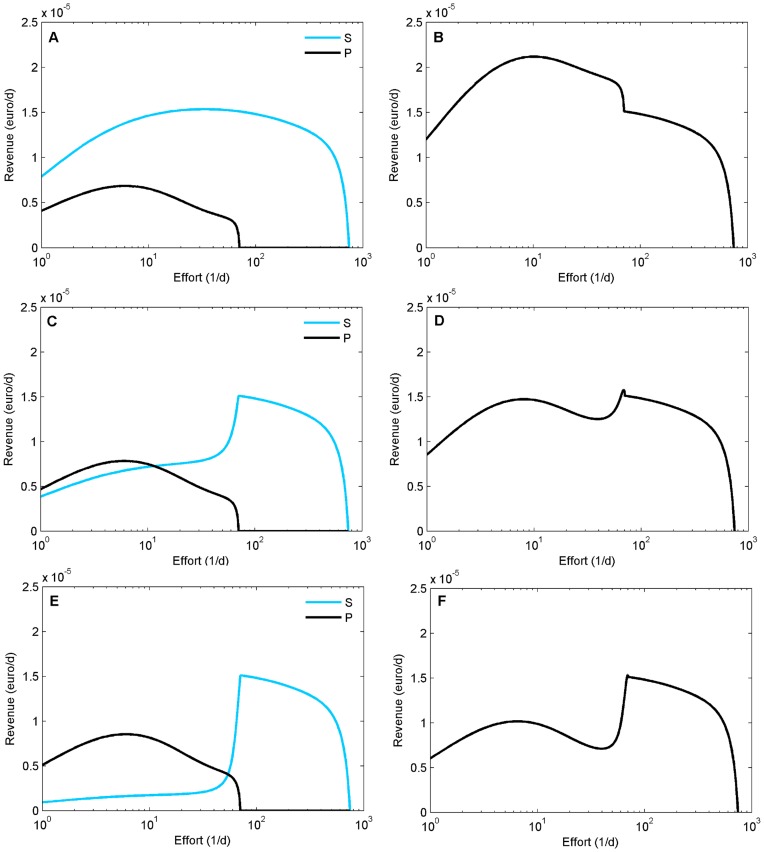
Revenue of the catches of sole and plaice as function of effort. Revenue of the catches of sole and plaice separately (A, C, E) and combined (B, D, F) as a function of increasing effort. Top row: ω = 0 (A, B); middle row: ω = 0.5 (C, D); bottom row: ω = 0.8 (E, F).

The revenue from the plaice and sole populations and the relationship between fishing intensity and revenue depends strongly on the resource overlap (Figure 5). Increasing effort from zero, revenue increases with increasing effort, because the increase in value per unit effort more than compensates for the decrease in adult density. This initial increase of revenue always occurs, but its magnitude in sole depends strongly on the degree of resource competition, since its adult density is strongly reduced at higher *ω* (compare Figure 5 A, C and E with increasing competition). At higher fishing effort, the adult biomass of each species becomes depleted to such an extent that higher effort does not substantially increase catches and hence revenue. In short, revenue always increases with harvesting effort at very low levels, and decreases with increasing effort at very high levels, irrespective of the degree of resource competition. In absence of resource competition (Figure 5 A), sole catches are always responsible for the majority of the revenue obtained from harvesting, but the revenue from sole is strongly reduced with increasing resource competition (compare Figure 5 A, C and E). The effort at which the combined revenue peaks (the maximum revenue from fishing is obtained) depends on the strength of interspecific resource competition (compare Figure 5 B, D and F). Without resource competition (Figure 5 B), the peak occurs at an effort where both species are present in the population, but already at ω = 0.5 (Figure 5 D), the global peak shifts to a harvesting effort where plaice is extinct, because sole has much higher revenue in absence of plaice. At higher resource competition this effect is even stronger (Figure 5 F). Additionally, a local maximum appears in the total revenue curve, caused by the peak of the revenue from the plaice population and the strongly reduced revenue from the sole population.

## Discussion

Our results show that including resource competition alters the effect of fishing effort on the population dynamics and persistence of species caught in indiscriminate or mixed fisheries. The effects of harvesting on the inferior competitor is influenced by competition, because the decrease in interspecific competition offsets the harvesting-induced mortality. An increase in fishing mortality may occur in response to increasing biomass of the inferior competitor. Because of the asymmetry in prices – sole is almost 10-fold more valuable than plaice – the increase in biomass of the inferior competitor due to a decrease in resource competition does have a profound effect on the revenue of harvesting. Increasing fishing mortality then leads to extinction of the superior species, which was already at low biomass, without loss of revenue based on the single, competitive inferior, species. With substantial resource sharing, the maximum revenue from harvesting is obtained at such a high harvesting level that plaice is harvested to extinction, while without resource sharing, the maximum occurs at a lower harvesting intensity, where both species can persist. An important consequence is that management geared towards extracting the maximum revenue from fish stocks may lead to extinction of the less valuable competitive superior species. It should be noted that the response to fishing mortality of populations regulated by competition may differ from those regulated by predator-prey interactions. Both interactions should therefore be considered in an ecosystem based management approach.

Our consideration of exploited populations linked by competition is highly relevant for the management of marine resources. Different species are often caught together in a fishery, so called mixed-fisheries. Currently, the management objectives for many of these fisheries are based on estimates of the maximum sustainable yield exploitation level. In a mixed fishery, the yields of different species could be consolidated by combining the yields in terms of monetary units. However, our results indicate that for systems where interspecific competition occurs between the species in the mixed fishery this maximum is obtained at fishing effort that result in extinction of one of the species. Link et al. [31] find similar results in a cross ecosystem comparison: overharvesting of stocks when inter-species and environmental interactions are not considered. Another important issue for fisheries management lies in the relationship between the biomass and the fishing effort. The fishing pressure on fish stocks is often measured by sequentially estimating the biomass of a fish stock. Combined with estimates of the amount of fish caught, the fishing mortality can be deduced. In classical stock assessment models, a decrease in the biomass indicates an increase in the fishing mortality (ceteris paribus). Here, we see that for part of the parameter space an increase in the biomass is actually linked to an increase in the fishing mortality. Hence, the competition among species could lead to incorrect interpretation of data on marine resource exploitation.

Our study shows that competition among harvested species can interfere with patterns of population response to exploitation derived from single-species based management. We use revenue in order to assess the effect of management and exploitation of the potential for exploitation of the combined stocks. Revenue is a better indicator of ‘exploitation potential’ than total biomass, due to the large market price asymmetry between the species. Despite our conversion of resource biomass to revenue, our aim is not to translate our ecological results to economic consequences. Such an economic analysis would require careful consideration of the costs associated with fishing, which is beyond the scope of this study. The bimodal curve of revenue over fishing effort that occurs when competition is included is due to the decrease in resource competition when plaice is nearly extinct. With high fishing effort, plaice, the superior competitor, no longer controls the shared resource. Fishing mortality in this case impacts as a top predator foraging on the competing species and changes the competitive outcome through ‘apparent competition, combined with exploitation competition [32]. Holt et al. [32] show that the exclusion of a ‘prey’ in such a system depends on predation mortality and resource availability. Changes in food availability due to external drivers such as eutrophication or climate change may further complicate the predictability of fisheries management in such systems.

The plaice population shows a stronger compensatory response to fishing mortality than sole does. The increase in biomass in the juvenile and large-juvenile stages with increasing effort is due to thinning which reduces resource competition and promotes reproduction for those adults still present [29], [30], [33]. While this effect at population level is due to the inclusion of stages, the difference in population response between plaice and sole is only partly due to the different (literature based) size ranges used for the species, while another part is due to the differences in other species-specific parameters (intake and catchability). Sole juveniles recruit to the large juvenile stage at larger sizes than plaice and this difference in sizes could influence the results because sole biomass will tend to concentrate less in the large juvenile stage than plaice biomass. As a result, sole is less affected by mortality from fishing. In order to test the influence of the stage boundaries on the result, the model was run with identical size ranges for the stages for both species, but not the species specific intake and catchability. The results qualitatively correspond to those reported in this work, including a stronger compensatory effect of plaice. Plaice is the superior competitor and is most prone to overfishing. Moreover, the bimodality in the revenue remains when using identical size ranges, but sole and plaice extinction occurs at higher values of maximum effort.

### Conclusion

Many fisheries indiscriminately target multiple fish species. Important implications for management of such fisheries emerge when the target species share the same food source, because resource competition among fish substantially complicates the relationship between fishing effort and fishing revenue. Management through separate quota for each species could in this situation lead to overfishing or even extinction of some species. Our findings highlight the need for an integrated multi-species management of fisheries that indiscriminately target multiple fish species.
